# Effect of three different forms of handling on the variation of aggression-associated parameters in individually and group-housed male C57BL/6NCrl mice

**DOI:** 10.1371/journal.pone.0215367

**Published:** 2019-04-12

**Authors:** Sinja Mertens, Miriam A. Vogt, Peter Gass, Rupert Palme, Bernhard Hiebl, Sabine Chourbaji

**Affiliations:** 1 Institute for Animal Hygiene, Animal Welfare and Farm Animal Behaviour and Virtual Center for Replacement—Complementary Methods to Animal Testing, University of Veterinary Medicine Hannover, Foundation, Hannover, Germany; 2 University of Heidelberg, Interfaculty Biomedical Research Facility (IBF), Heidelberg, Germany; 3 University of Heidelberg, Central Institute of Mental Health (CIMH), Mannheim Faculty, Heidelberg, Germany; 4 University of Veterinary Medicine Vienna, Unit of Physiology, Pathophysiology and Experimental Endocrinology, Vienna, Austria; Harvard University Faculty of Arts and Sciences, UNITED STATES

## Abstract

Mice are social animals hence group-housing of mice is preferred over individual housing. However, aggression in group-housed male mice under laboratory housing conditions is a well-known problem leading to serious health issues, including injury or death. Therefore, group-housed mice are frequently separated for welfare reasons. In this study, we investigated the effect of 3 different handling methods (tail, forceps, tube) in 2 different housing conditions (single vs. group) on the variance of aggression-associated parameters in male C57BL/6NCrl mice over 8 weeks. Blood glucose concentration, body weight, body temperature, plus number and severity of bite wounds and barbering intensity in group-housed mice were recorded. An assessment of nest complexity was also performed weekly. Feces were collected in week 3 and 7 for analysis of corticosterone metabolites. We also monitored the level of aggression by recording the behavior of group-housed animals after weekly cage cleaning. An open field test followed by a social novel object test, a light/dark box test, a hotplate and a resident-intruder test were performed at the end of the 8-week handling period. Post-mortem, we assessed organ weights. We found that forceps-handled mice, independent of the housing condition, had significantly higher levels of stress-induced-hyperthermia and enhanced aggression after cage cleaning, and they performed worse in the nest complexity test. In addition, handling male mice by the tail seems to be most effective to reduce aggressiveness after transferring animals into new cages, thereby representing an appropriate refinement.

## Introduction

Before being used in experimental procedures, laboratory mice spend most of their lives in their home cages. According to European Union legislation, maintenance procedures for rodents must modify environment and handling to the behavioral and physiological needs of animals [1]. A major challenge in this context is that there is not much literature systematically addressing which measures and procedures cause stress or well-being, respectively, which is an essential prerequisite both for the well-being of experimental animals and reliable *in vivo* research [2].

Housing social animals in groups is required where applicable. However, group-housing of male mice is a concern in most animal facilities due to potential welfare concerns. Despite daily animal observations, aggression may not be adequately monitored since it can arise spontaneously resulting in pain, injury or death of the animals [3]. Besides affecting welfare and numbers of animals needed, aggression may result in serious problems with data validity [3].

While there are sophisticated refinement approaches with regard to housing conditions, such as by providing enrichment, it is necessary to thoroughly evaluate such measures, since additional cage equipment may also induce aggression as demonstrated by Howerton *et al*. [4]. Similarly, Marashi *et al*. showed negative effects of housing environment with regard to related physiological and immunological stress parameters [5]. Such findings indicate the importance of adequate awareness not only for experimental design, but also in regards to the animals’ history.

Besides housing, animal handling is a regular necessity in the animal facility. Handling is the most common procedure experienced by laboratory mice because it is necessary for routine husbandry (*i*.*e*. cage cleaning) and research procedures (*i*.*e*. injections or blood sampling). Due to pragmatic reasons, handling usually takes place in a context in which the animals anyhow experience stress, *i*.*e*. when disturbed in their inactive phase [6]. It is necessary to consider all potential interacting stressful factors for any kind of refinement program–handling being one of those. The distress of male laboratory mice is attributed to housing and experimental procedures for which handling is necessary, and handling related distress in the pre-experimental history of the animals represents aversive experiences and may exert long-lasting effects [7]. Conversely, animal distress may be reduced by certain procedures implemented in handling, thus taming their anxiety [8].

Bearing in mind that many neurobehavioral disorders are investigated in male mice [9], it is necessary to address how refinements of housing and handling may influence male aggressive responses. One paradox here is that males have been historically preferred over females due to the perception of “better” data quality due to less hormonal variability [9]. Whether aggression and dominance structures in males may affect inconsistency of data is mostly neglected. This is interesting, because males, but not females, housed in unisex groups demonstrate several behavioral [10] and physiological alterations [11].

In our study we studied the effects of routinely conducted handling by tail in the context of cage changing and compared this procedure with forceps and tube handling. As readout we focused on behavioral effects which were assessed in a behavioral test battery comprising tests for exploration (open field test), emotional states (light/dark box) and aggression (resident-intruder test). This was complemented by health monitoring (fur state) and assessment of clinical parameters, *i*.*e*. blood glucose assessment, physiological measures of stress, *i*.*e*. stress-induced hyperthermia, fecal corticosterone metabolites (FCM) as well as final organ weight determination. To analyze handling effects on animal welfare, we conducted a nest building assay (after Deacon *et al*. [12, 13]). To evaluate potential consequences of handling on agonistic behaviors, single-and group-housed males were examined with regard to data variability by looking at the standard deviation of means. We hypothesized that signs of stress and aggression should be reduced in both single- and group-housed mice if the handling condition is less stressful to the mice. Additionally, we hypothesized stress would lower the scores in the nest building test, the well-being parameter, in which we observed the mice’s nest building performance over time.

## Materials and methods

### Ethics statement

The study was conducted according to the guidelines of the German Animal Welfare Act and was approved by the Karlsruhe State Authority (permit number: G-154/17).

### Animals

72 male C57BL/6NCrl mice (Charles River, Sulzfeld, Germany) arrived at the facility at the age of 3 weeks. The C57BL/6 strain is frequently used and one of the most common background strains for transgenic mouse models [14]. All mice were housed in Macrolon II cages (370cm^2^, Tecniplast, Milan, Italy), provided with aspen wood bedding (ABEDD LTE-001, Lab & Vet Service, Vienna, Austria) and a nestlet (Plexx B.V, AB Elst, The Netherlands). Tap water and food pellets (Rod 16-A LasVendi, Soest, Germany) were provided *ad libitum*. The animal room had a controlled temperature (21°C), photoperiod (reversed 12/12 h light/dark cycle: lights on between 21:00–09:00 h) and relative humidity (50–60%). The hygienic status was specific pathogen-free (SPF) according to Federation of European Laboratory Animal Science Association’s (FELASA) recommendations [15].

Out of all mice, 54 mice were divided into groups of 3 (18 cages) and the remaining 18 mice were housed individually for a total of 36 cages. Single (n = 18) and group-housed (n = 54) mice were arbitrarily allocated into three subgroups (transferring mice by picking them up by their tails with gloved hand using thumb and index finger (latex powder-free gloves, sempercare, premium by sempermed), single tail SH/ group tail GH, n = 6/18; by tube, single tube ST/ group tube GT, n = 6/18 or by forceps, single forceps SF/ group forceps GF, n = 6/18) according to the order they were unpacked. All animals were marked on the tail with a black waterproof marker (renewed weekly) and in addition by ear punches on arrival. In the group-housed condition only the mouse marked on arrival as number three was exposed to behavioral testing.

Interaction-partners for behavioral testing (sNO and RIT testing) were 8 weeks old male mice of the C3H/HeJRj inbred strain (Janvier, Laval, France), which arrived at the facility 1 week before being used for the resident-intruder test (RIT). This strain was selected because of its brown color (easier to distinguish from the black C57BL/6NCrl mice in the RIT) and due to their even tempers (personal communication with commercial breeders). They were housed under the same abiotic conditions as the experimental subjects, but in a separate room which had no reversed light/dark cycle (lights on between 09:00–21:00 h).

### Experimental setup

**Fig 1:**
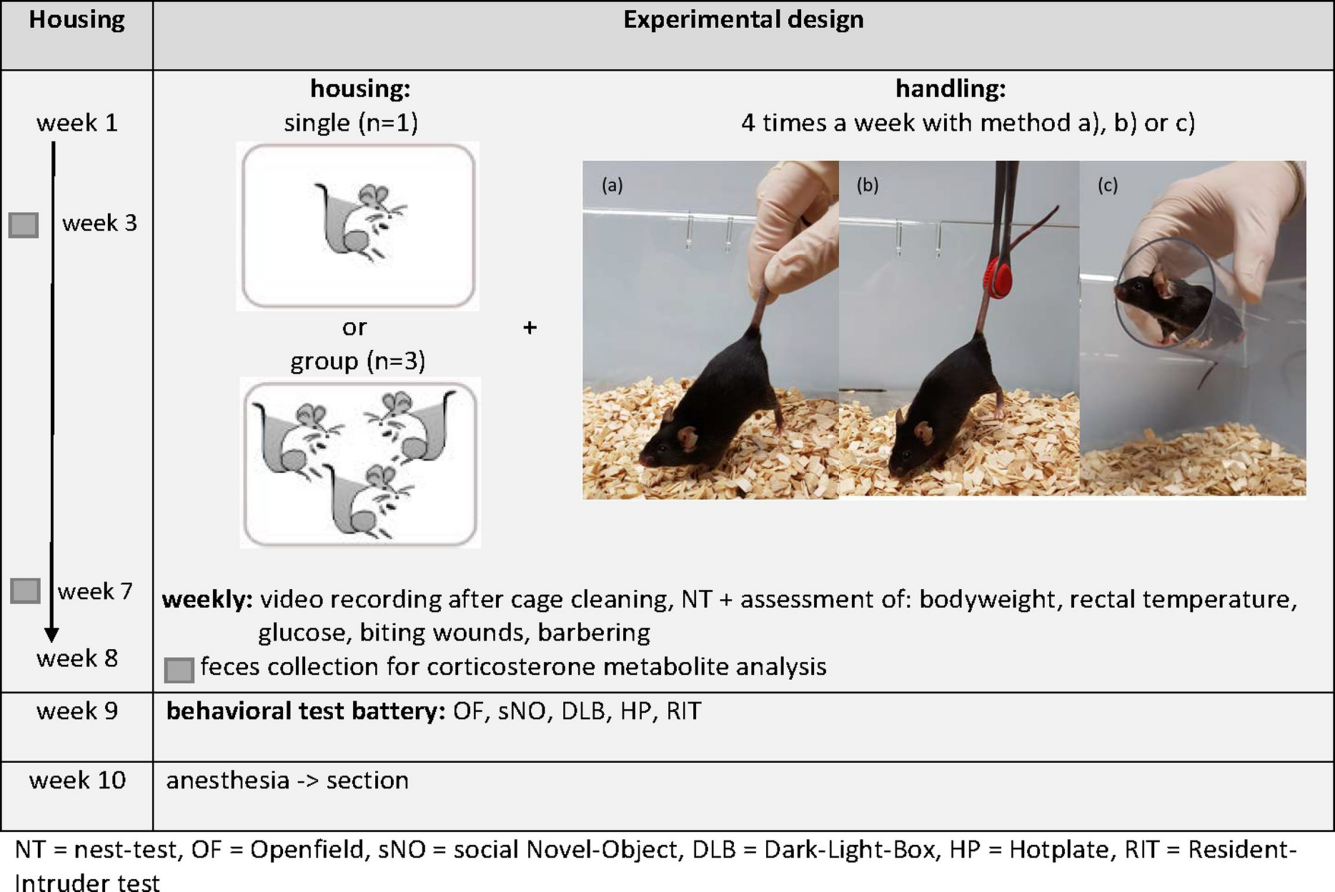
Experimental design. Overview of the procedures for single- and group-housed cohorts: (a) = tail; (b) = forceps; (c) = tube. Behavioral analysis (except the NT) of mice started at week 9 and lasted until the animals were euthanized at 10 weeks.

Experiments and handling were conducted during the dark phase, as illustrated in **Fig 1.** Mice were handled 4 times a week by one female experimenter by the assigned handling method, *i*.*e*. tail handling, forceps handling (Mouse Holding Forceps with Replaceable Tips, FST, Heidelberg, Germany) or tube handling (polycarbonate mouse handling tube, Datesand Group, Manchester, United Kingdom). Mouse cages were opened and mice were lifted one by one via their allocated handling method, placed on the wire lid, and then returned to their home cage. As the first 2 weeks of handling served as acclimation, the data collected in this period were not used for statistical analysis.

Cage cleaning and the nest building test were performed weekly on the same day (except before the RIT). Immediately after transferring the group-housed mice into clean cages, their behavior was recorded by a camera (Ikegami Digital) for 20 min.

One day after cage cleaning, mice were weighed, their fur was examined for wounds or barbering signs according to a standardized score sheet (S1 Fig). Tail blood was taken by puncturing the vein with a cannula for glucose determination and body temperature was measured by gently inserting a rectal thermometer 1cm twice, 30 min apart, to determine stress-induced hyperthermia (SIH). To minimize circadian influences on behavior, the starting point in the order of mice being handled and the order of cages cleaned, recorded and measured was changed weekly. All animal care and testing were carried out by the same person. Six days after cage cleaning (weeks 3 and 7) feces were collected for FCM analysis.

To investigate potential handling-, and housing-induced aggression, one mouse (number 3) out of each cage was subjected to 4 behavioral tests at the age of 11–12 weeks: The open field test (OF) followed by a social novel-object test (sNO), light/dark box test (LDB), hotplate (HP) and RIT. On each testing day mice were transferred to the testing room 25 min before behavioral testing for acclimatization, and only one test was conducted sequentially per day.

### Behavioral analysis

#### Nest building test

A standard nestlet (Plexx B.V, AB Elst, The Netherlands) and approximately 0.5 g of the old nesting material (to decrease aggression in male mice [3, 16]) was transferred into the clean cage at the day of cage change. The nest quality was analyzed after 5 and 24 h using a scoring system modified from Deacon [13]. Score 1: nestlet untouched, score 2: nestlet largely untouched (> 90% intact), score 3: nestlet mostly shredded (< 50% intact nestlet), score 4: identifiable, but flat nest (> 90% torn up), score 5: partial cup, score 6: full dome.

#### Behavioral scoring of aggression-associated parameters

Only group-housed mice were weekly recorded after cage cleaning for 20 min at 35 lux by a camera positioned above the home cages. We assessed the i) attack latency, ii) the first attacks’ duration and iii) the number of attacks from the video. In accordance to Garner *et al*. [17] an attack was defined as the rushing and leaping at a partner with bites and kicks.

#### Open field and social novel-object test

Mice were examined in an open field test (OFT) for measurement of locomotion, anxiety and exploration [18] for 2 days between 10.00 a.m. to 1.30 p.m. The animal was placed using its assigned handling method into a square, black OFT arena (50 x 50 cm^2^) placed on an infrared light surface illuminated with 25 lux from above. Up to 3 mice were tracked simultaneously in separate arenas for 10 min and filmed by a camera from above. ‘Velocity’, ‘distance moved’ and the time spent in defined areas of the OF, *i*.*e*. ‘center time’ were evaluated [19]. Subsequently a social novel-object (sNO) test was conducted. After 10 min of habituation to the empty OFT arena, an unfamiliar C3H mouse in a small grid-box (7 cm x 7 cm x 8 cm) was placed in the center of the arena (10 cm distance to walls), representing a social novel object. By evaluating the ‘latency’, ‘the time’ and ‘the number of approaches’ (an approach = mouse is nearing the grid box in close vicinity with head turned in the direction of the box) in the sNO, we measured exploration and social behaviors. Finally, number of fecal boli produced during the OFT as well as the sNO test was counted as an indicator of emotionality [20]. The videos were processed within Noldus EthoVision 4.0 (Noldus Information Technology, Wageningen, The Netherlands).

#### Light/dark box test

A Light/dark box test was performed to examine anxiety-like behaviors. The mouse was placed into the dark compartment (22.5 x 22.5 cm^2^, approximately 1 lux) which was connected by an aperture with the light compartment (31.5 x 22.5 cm^2^, approximately 600 lux). Number and latency of exits into, as well as time spent in the lit compartment was recorded for 5 min by a camera positioned above the box as previously described [21]. An exit was defined as the placement of all four paws into the light chamber [22].

#### Hotplate test

To test a mouse’s reaction to thermal pain, it was placed in the center of a clear Plexiglas cylinder (20 cm diameter) atop a hot plate (Ugo Basile Hot/Cold Plate 35100, Ugo Basile, Gemonio, Italy) which was maintained at 53 ± 0.3°C. The time until the mouse first lifted its hind paw followed by clear paw flinching or licking movements was recorded and the animal was then removed from the plate. To avoid possible tissue damage the cut off time for an animal to react was set at 45 s [23].

#### Resident-intruder test

Using a RIT, mice were tested towards a stranger to monitor aggressive-like behaviors [24, 25]. 12 days prior to the test the home cage was not changed to maintain territoriality, which is strongly dependent on the presence of olfactory cues in the bedding [26]. The test began when the intruder (unfamiliar C3H male)) was placed in the resident cage. Interactions between the test mouse and the intruder were recorded (camera from above, 3 lux) for a period of 10 min.

Behavior was scored from video [17] (focusing only on the behavior of the tested mouse). The duration of threat behavior (tail rattling, thrust, mounting) and aggressive behavior (attack latency, boxing, aggressive bite, attack, fighting, chase) were measured. Mice that did not fight during the test were excluded from the statistical outcome. Severe injuries of the C3H mouse would have led to an immediate termination of the test.

### Clinical parameters

Barbering and biting wounds were recorded throughout the study by means of a standardized score sheet (S1 Fig).

#### Body weight

The body weight of each mouse was measured once a week.

#### Blood glucose

Weekly blood glucose levels were measured prior to determination of body temperature. Mice were placed on a cage top using their assigned handling method. Blood samples were obtained from the tail vein by puncture. Assessment of blood glucose level was done by means of a glucometer (Medisana MediTouch 2, Promed GmbH, Germany).

#### Body temperature

Body temperature was measured as an indicator of stress-induced hyperthermia (SIH) by using a rectal thermometer (Testo 108, Testo SE & Co. KGaA, Lenzkirch, Germany + MLT1404 Rectal Probe, ADInstruments Ltd, Oxford, United Kingdom) which was inserted, after dipping it into a lubricant (Vidisic, Bausch + Lomb, Germany), for a length of 1 cm into the rectum of the mice at time point T_1_ (min) and time point T_2_ (min, T_1_ + 30 min). This required brief restraint by the tail: After being placed with their determined method on a wire top of an empty cage, their tail was hold up by the researchers’ hand to insert the rectal probe, hence all animals were touched on their tails. The difference, ΔT (= T_2_ –T_1_) is the measure for SIH, which is defined as a relative short lasting body temperature elevation in response to stress [27]. Only temperature differences at point ΔT of more than 0.5°C were defined as SIH.

#### Feces collection and FCM analysis

Stress hormone levels of mice were monitored non-invasively by measuring fecal corticosterone metabolites (FCM). Feces collection from the 3^rd^ and 7^th^ week was performed at day 6 after cage cleaning around 2 pm. Mice were placed individually in empty Macrolon Type II cages (370cm^2^, Tecniplast, Milan, Italy) for a maximum of 50 min. The sampling occurred in a limited time frame as corticosterone concentration has a circadian rhythm, so once the required number of fecal boli was reached (± 6 droppings), the mouse was returned to the home cage.

Feces were stored in polypropylene tubes at -20°C. FCM were extracted in accordance with Palme *et al*. [28]: Feces were dried for 1 h at a temperature of 70°C and homogenized. An aliquot of 0.05 g was extracted with 1 ml of 80% methanol and the extracts stored at -20°C. Later they were analyzed using a 5α-pregnane-3β,11β,21-triol-20-one enzyme immunoassay [29, 30].

#### Organ weights

Mice were deeply anesthetized and euthanized i.p. with ketamine 195 mg/kg body weight (Bremer Pharma GmbH, Warburg) and xylazine 30 mg/kg body weight (Ecuphar GmbH, Greifswald). Thymus, spleen, adrenal glands, seminal vesicles, and testes were dissected and weighed.

#### Statistical analysis

All statistical analyses were performed using SPSS software, version 25 for Mac (IBM). Parametric and non-parametric tests were utilized depending on data distribution (Q-Q plots or Shapiro-Wilk test). For parametric data, two-way analysis of variance (ANOVA) were carried out with ‘housing’ and ‘handling’ as factors and the Tukey test as post hoc test. An intra-group comparison of the body weight and blood glucose was carried out by using a repeated measurement analysis, in which the factor ‘housing’ and ‘handling’ was added. Non-parametric data was analyzed by the chi-square statistics, Mann-Whitney-U-Test, Kruskal-Wallis-Test and the Friedman-Test. Additionally, we calculated the standard deviation of all values, serving as a measure of variation observed in the data. Statistical significance of variation was evaluated by a Levene test. P ≤ 0.05 was considered statistically significant. * indicates p ≤ 0.05, ** indicates p ≤ 0.01 and *** indicates p ≤ 0.001.

## Results

Table 1 illustrates significant and non-significant findings regarding the factor ‘housing’ and ‘handling’.

**Table 1:** Statistical results concerning the factors ‘housing’ and ‘handling’.

				Effects/ interactions			Post hoc test (Tukey)			Effect size (Cohen's d)			
	Test	Parameter	Statistical test	Housing	Handling	Housing x handling	Hand vs. forceps	Hand vs. tube	Forceps vs. tube	Housing	Hand vs. forceps	Hand vs. tube	Forceps vs. tube
**Clinical parameters**	Body weight	weight, week 8 (g)	ANOVA	*p = 0.039, F = 4.43, df = 1	n.s.	n.s.	n.s.	n.s.	n.s.	-0.58	-0.07	0.02	0.09
	Temperature difference	temperature difference, week 7 (°C)	ANOVA	*p = 0.046, F = 4.14, df = 1	n.s.	n.s.	n.s.	n.s.	n.s.	0.60	-0.49	-0.45	0.04
		temperature difference, week 8 (°C)		n.s.	**p = 0.009, F = 4.99, df = 2	n.s.	**p = 0.01	n.s.	p = 0.059	0.11	-0.82	-0.21	0.67
	Stress induced hyperthermia	temperature difference, week 8 (°C)	Chi-squared	n.s.	*p = 0.028		*p = 0.019	n.s.	*p = 0.019				
	Blood glucose	Blood glucose, week 8 (mmol/L)	ANOVA	n.s.	n.s.	**p = 0.005, F = 6.5, df = 2	n.s.	n.s.	n.s.	-0.13	0.33	0.36	0.09
	Fecal corticosterone metabolites	corticosterone, week 7 (pg/well)	ANOVA	**p = 0.005,F = 8.98, df = 1	n.s.	n.s.	n.s.	n.s.	n.s.	-1.00	0.20	-0.50	-0.56
		corticosterone, week 7 (ng/0.05 g feces)		**p = 0.005, F = 8.98, df = 1	n.s.	n.s.	n.s.	n.s.	n.s.	-1.00	0.20	-0.50	-0.56
		corticosterone, week 7–3 (pg/well)	ANOVA, repeated measurements	**p = 0.002, F = 10.79, df = 1	n.s.	n.s.	n.s.	n.s.	n.s.				
		corticosterone, week 7–3 (ng/0.05 g feces)		**p = 0.002, F = 10.77, df = 1	n.s.	n.s.	n.s.	n.s.	n.s.				
**Behavioral analysis**	BS of aggression-associated parameters	cages showing aggression, week 5–8, attack yes/no	Chi squared	/	**p = 0.002		**p = 0.004	n.s.	*p = 0.02				
	Nest-test	quality, week 3, 5 h scores	Mann-Whitney-U or Kruskal-Wallis	*p = 0.025	n.s.		n.s.	n.s.	n.s.				
		quality, week 4, 5 h scores		**p = 0.01	n.s.		n.s.	n.s.	n.s.				
		quality, week 6, 24 h scores		**p = 0.01	n.s.		n.s.	n.s.	n.s.				
		quality, week 8, 24 h scores		*p = 0.044	p = 0.061		*p = 0.033	n.s.	n.s.				
		quality, week 3–8, 5 h scores		***single: p < 0.001 ***group: p < 0.001	***hand: p < 0.001 *forceps: p = 0.037 ***tube: p = 0.001	*SH p = 0.011 SF: n.s. *ST: p = 0.013 ***GH: p = 0.001 GF: n.s. GT: p = 0.068							
		quality, week 3–8, 24 h scores		*single: p = 0.027; group: p = 0.102	hand: p = 0.32; forceps: p = 0.145; *tube: p = 0.049	SH: n.s. SF: n.s. *ST: p = 0.043; GH: n.s. GF: n.s. GT: n.s.							
	Openfield	distance to walls 0–10 min (cm)	ANOVA	**p = 0.006, F = 8.76, df = 1	n.s.	n.s.	n.s.	n.s.	n.s.	-0.89	-0.15	0.54	0.69
		time in center 0–10 min (s)		**p = 0.0011, F = 7.2, df = 1	n.s.	n.s.	n.s.	n.s.	n.s.	0.93	0.12	-0.38	-0.56
	Social Novel-Object test	distance moved 0–5 min (cm)	ANOVA	n.s.	n.s.	*p = 0.035, F = 3.75, df = 2	n.s.	n.s.	n.s.	-0.02	0.15	0.51	0.39
		velocity 0–5 min (cm/sec)		n.s.	n.s.	*p = 0.036	n.s.	n.s.	n.s.	-0.05	0.15	0.50	0.37
		time in center 0–10 min (s)		*p = 0.043, F = 4.4, df = 1	n.s.	n.s.	n.s.	n.s.	n.s.	-0.70	0.15	-0.03	-0.15
	Light/dark box test	exit latency (s)	Mann-Whitney-U or Kruskal-Wallis	n.s.	*p = 0.031		n.s.	**p = 0.007	n.s.				
		tail rattling (s)	Mann-Whitney-U or Kruskal-Wallis	*p = 0.036	n.s.		n.s.	n.s.	n.s.				
		aggressive bite (s)		*p = 0.043	n.s.		n.s.	n.s.	n.s.				
		attack (s)		*p = 0.024	n.s.		n.s.	n.s.	n.s.				
		fighting (s)		*p = 0.016	n.s.		n.s.	*p = 0.038	n.s.				

n.s. = no significance. Identification of overall effects of the factors 'handling' and 'housing' as well as interaction effects. Asterisks indicate the level of significance (* p ≤ 0.05; ** p ≤ 0.01; *** p ≤ 0.001). Data is split in 'clinical parameters' and 'behavioral analysis' for better overview. Non-parametric tested parameters are highlighted in grey. The effect size (Cohen’s d) was calculated for normal distributed data analyzed with ANOVA. Parameters not appearing in this table revealed non-significant results.

### Barbering and biting wounds

No bite wounds were visible on external exam during the experiment. In week 8, 2 mice of a GH-cage showed hair loss on their heads, which we attribute to barbering behavior of the third mouse in the cage.

### Nest building test

Immediate nest building improved over the course of the experiment (3^th^ - 8^th^ week) in both housing conditions (single housed: Chi^2^ (5) = 33.3, p < 0.001; group housed: Chi^2^ (5) = 29.4, p < 0.001) and in all animal cages. Significant results were detected for the groups SH (Chi^2^ (5) = 14.9, p = 0.011), ST (Chi^2^ (5) = 14.4, p = 0.013) and GH (Chi^2^ (5) = 21.5, p = 0.001) measured by the Friedman-test. When nest quality was assessed after 24 h, only singly-housed animals improved their nest building performance over the course of the experiment (Chi^2^ (5) = 12.6, p = 0.027). This only applies for singly-housed, tube-handled mice in a statistically significant way (Chi^2^ (5) = 11.4, p = 0.043).

**Fig 2:**
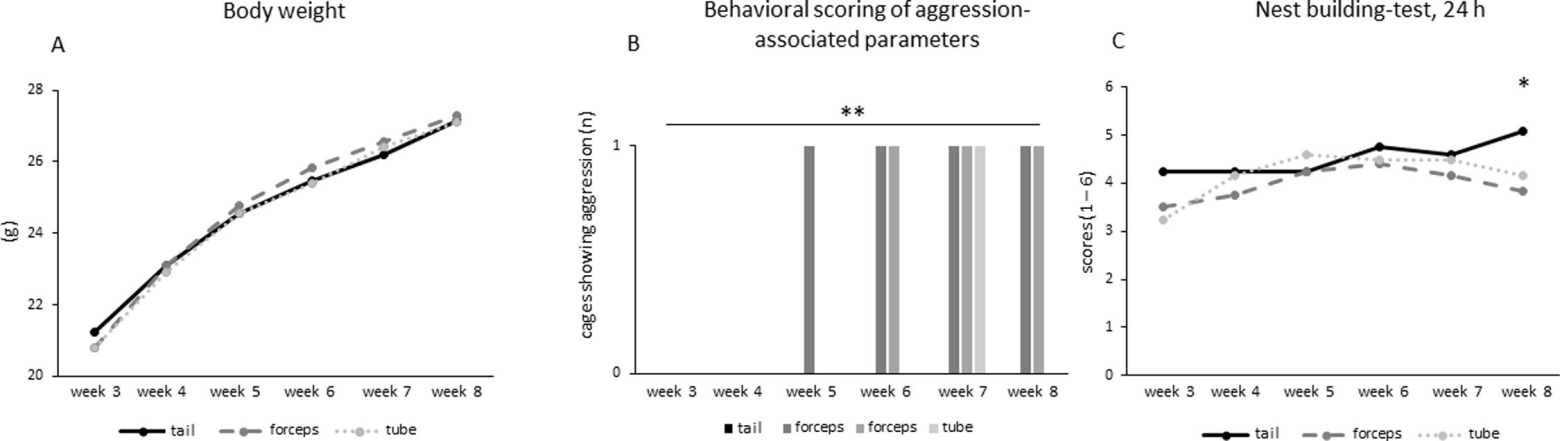
Development. A) Nest building test, 24 h (n of cages = 36, 24 mice/handling method). A significant overall effect (p < 0.05) revealed regarding the factor ‘handling’. Furthermore, a significant difference in the nest building performance is seen by the last week of housing and handling, indicating the role of experience. B) Behavioral scoring of aggression-associated parameters, (n = 54, 18/handling method). There was a significant overall effect concerning the number of attacks: forceps-handled group-housed mice had an increase in the number of attacks compared to the other handling methods (p < 0.01). C) Body weight, (n = 72, 24/handling method). No difference in the development was found regarding the factor ‘housing’ or ‘handling’. Asterisks indicate the level of significance (* p ≤ 0.05; ** p ≤ 0.01; *** p ≤ 0.001).

24 h scoring in the 8^th^ week, measured with a non-parametric-test for independent samples, revealed an overall effect for the factor ‘housing’ (z = -2.01, p = 0.044). Post hoc testing showed a significant difference (U = 35, p = 0.033) between tail-handled animals and forceps-handled animals **(Fig 2A)**. No significant effects after 5 h in week 8 were detected.

### Behavioral scoring of aggression-associated parameters

The first aggressive behavior after cage cleaning appeared in 8 weeks old group-housed mice handled with forceps (GF) in the 5^th^ week. Using the chi squared test on proportions of cages displaying aggression there was a significant overall effect concerning the handling methods (Chi^2^ (2) = 12.09, p = 0.002), (**Fig 2B)**. Tail-handled animals showed no aggression at all, 1 out of 6 tube-handled cages only once (7^th^ week) and 2 out of 6 forceps-handled cages showed aggression from 5^th^ and 6^th^ week until end of recording.

### Body weight

Animals gained weight over time regardless of housing condition. At the conclusion of the experiment, an effect for the factor ‘housing’ was seen. Singly-housed mice were significantly lighter than group-housed mice (F_(1,70)_ = 4.43, p = 0.039). No differences in weight could be attributed to ‘handling’, however. **(Fig 2C)**. Additionally, variation differences were investigated for both factors but were not significant.

### Body temperature

**Fig 3:**
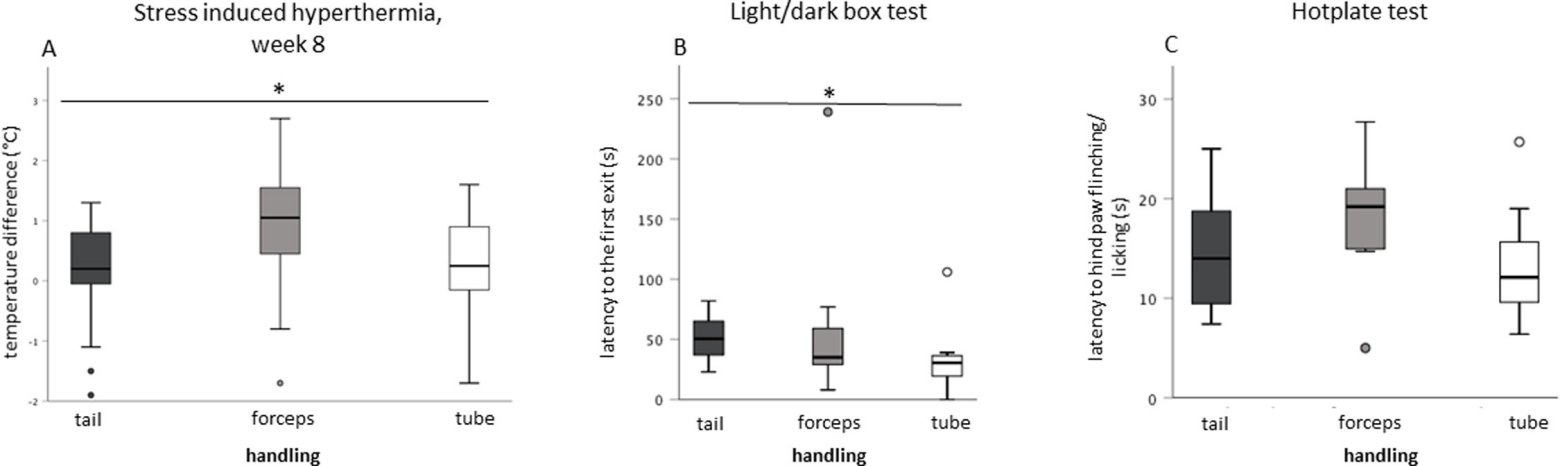
Handling effects. A) Stress induced hyperthermia, (cages n = 36, 24 mice/handling method). Effect of handling treatment on the SIH in 8^th^ week. A higher temperature difference was found for forceps-handled animals compared to tube- and tail-handled mice (p < 0.05). B) Light/dark box test, (n = 36, 12/handling method). Evaluation of anxiety behavior regarding the factor ‘handling’ in a LDB test. Tube-handled mice had diminished anxiety behavior represented by the latency to the first exit. C) Hotplate test, (n = 36, 12/handling method). Asterisks indicate the level of significance (* p ≤ 0.05; ** p ≤ 0.01; *** p ≤ 0.001).

There was a significant overall effect only in the 7^th^ week for the factor ‘housing’ (Chi^2^ (1) = 5.04, p = 0.025) with singly-housed mice showing increased hyperthermia. In the 8^th^ week, a significant difference in the factor ‘handling’ (Chi^2^ (2) = 7.13, p = 0.028) was noted. Post hoc testing revealed a significant difference between forceps-handled and tube-handled mice (Chi^2^ (1) = 5.49, p = 0.019) or tail (Chi^2^ (1) = 5.49, p = 0.019) **(Fig 3A)**. Furthermore, forceps-handled males had the greatest variation in temperature difference (tail, 0.2 ± 0.8; forceps, 0.9 ± 1; tube, 0.3 ± 0.8; mean ± SD) confirmed by Levene testing (tail vs. forceps: p = 0.026; tube vs. forceps: p = 0.026).

### Blood glucose

Neither housing nor handling influenced blood glucose levels overall. In the 8^th^ week, the interaction between housing and handling was significant (F_(2,36)_ = 6.5, p = 0.005). Tail-handling in singly-housed mice led to the lowest, whereas in group-housed animals this handling condition evoked the highest blood glucose levels. For forceps-handled mice the blood glucose levels under group- and single housing conditions were most similar. Tube-handling in singly-housed mice revealed the highest and in group-housed animals the lowest blood glucose levels.

### Light/dark box test

Anxiety-like behaviors analyzed in the LDB were altered depending on handling conditions. This was especially evident in the ‘latency to the first exit’ (Chi^2^ (2) = 6.92, p = 0.031). Tail-handled mice entered the lit compartment significantly later compared to tube-handled mice with post hoc testing (U (1) = 26.5, p = 0.007), **(Fig 3B)**.

### Hotplate test

Mouse reaction time on the hotplate was not dependent on the handling condition (F_(2,33)_ = 3.08, p = 0.059), **(Fig 3C)**.

### Open field and social novel-object test

**Fig 4:**
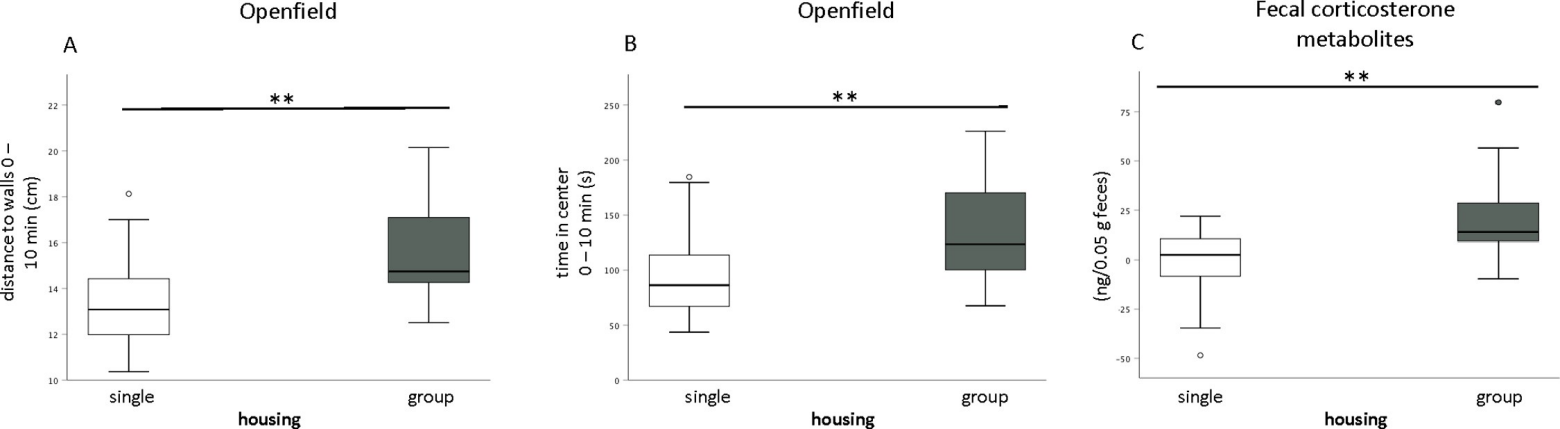
Housing effects. A-B) Open field, (n = 36, 18/group). Behavior of C57BL/6N mice in the OF. Group-housed mice stayed more distant to the walls (A), consequently they spent more time in the center (B). C) Fecal corticosterone metabolites (FCM), week 7–3, (n = 36, 18/group). Group-housed mice had higher FCM levels (** p = 0.002). Asterisks indicate the level of significance (* p ≤ 0.05; ** p ≤ 0.01; *** p ≤ 0.001).

Open field: Locomotion in the OF was significantly altered by the factor ‘housing’ concerning the parameters ‘distance to walls 0–10 min’ (F_(1,34)_ = 8.76, p = 0.006) and the ‘time in center 0–10 min’ (F_(1,34)_ = 7.2, p = 0.011). Group-housed mice compared to single-housed mice spent more time in the center, hence achieved greater distances to the walls **(Fig 4A and 4B).**

Social novel-object: Tukey post hoc testing revealed that tube-handled animals spent significantly more time (t = 2.47, p = 0.049) near the interaction partner compared to forceps-handled animals. Depending on the housing condition and the handling method mice differed in the first 5 min in their outcome in the parameters ‘distance moved’ (F_(2,30)_ = 3.75, p = 0.035), ‘velocity’ (F_(2,30)_ = 3.71, p = 0.036). Additionally, group-housed animals spent significantly more time in the center (F_(1,34)_ = 4.4, p = 0.043) compared to singly-housed ones.

### Fecal corticosterone metabolites (FCM)

Handling had no significant effect on FCM concentrations (ng/0.05 g feces) when analyzed with a one-way ANOVA after we calculated the changes relative to the absolute baseline value at week 3 (F_(2,33)_ = 0.24, p = 0.791). The same applies for absolute values measured in week 3 (F_(2,33)_ = 2.17, p = 0.13) or 7 (F_(2,33)_ = 1.42, p = 0.256). A bifactorial ANOVA revealed significant differences between group and individual-housed animals (F_(1,34)_ = 10.77, p = 0.002) **(Fig 4C)**. No difference regarding absolute FCM values in week 3 (F_(1,34)_ = 0.76, p = 0.392) could be observed, whereas in week 7 a significant difference existed (F_(1,34)_ = 8.98, p = 0.005).

### Resident-intruder test

Of the 36 mice (6 mice of each condition; as stated above only one defined mouse of the group-housed mice was participating in the behavioral tests) used in this study, 21 (58%) were aggressive towards an intruder male and included in the statistical analysis. Single mice showed in general more aggressive behavior when analyzed with a Mann-Whitney-U-Test for independent samples, reflected in detail by: significantly more events of tail rattling (U = 25, p = 0.036), number of aggressive bites’ (U = 26.5, p = 0.043) as well as attacks’ (U = 23, p = 0.024) and ‘fighting’ (U = 21.5, p = 0.016). Handling conditions did not influence these behaviors significantly. A high variation for forceps-handled animals’ data was notable for the parameter ‘fighting’ (tail, 24.7 ± 15.7; forceps, 58 ± 56.1; tube, 42.4 ± 13.3; mean ± SD), confirmed by the Levene test (tail vs. forceps: p = 0.007; forceps vs. tube: p = 0.004).

### Organ weights

All organs were removed by the same animal technician. Neither the housing condition nor the handling procedure significantly affected mouse organ weights.

### Discussion

Our results indicate that forceps handling has a negative impact on aggression-associated parameters. Forceps-handled mice had visible deficits in the well-being parameter represented by the nest building test and an increase in the number of attacks after cage cleaning, higher (stress) reaction in the stress induced hyperthermia and less pain perception on the hotplate. Contrary to our expectations, tube handling only affected anxiety-like behavior and offered no advantages over tail-handling in all other investigated parameters.

In the literature tunnel- or tube-handling is praised as a non-aversive method that reduces anxiety-like behavior as well as optimizes the reliability of behavioral testing in laboratory mice [8, 31–33]. In our study, the tube-handled mice were the fastest to exit the dark compartment in the LDB test, which indicates they experienced less anxiety, consistent with the previous findings [31, 34]. In contrast, the performance of mice picked up by the tail with a gloved hand was the poorest reinforcing the conclusion by Hurst *et al*. that this traditional way of mouse handling causes stress and anxiety [8]. Contrary to the outcomes reported in Gouveia *et al*. [31], the handling method did not have a strong influence on the general exploratory behavior when tested in the OF. In the sNO test, tube-handled animals did spend more time with the newly introduced interaction partner than those lifted by the tail. Hence, one may assume their curiousness/social drive to be greater than their anxiety. No further changes in behavior associated with tube-handling were observed. Nest building performance of tube-handled mice over the time did not differ from tail-handled animals after 5 h, whereas they performed better than forceps-handled animals. After 2 months of housing the scores after 24 h were not as good as in tail-handled animals, but still better than in forceps-handled mice. In stress-related parameters such as the SIH, tube- and tail-handled animals had similar results.

There are several possible explanations for the restricted outcome of the actual predicted advantage of tube-handling: First, for experimental testing, *i*.*e*. to gain blood, mice in our study were restrained once a week on a wire cage lid by hand, while Hurst and colleagues [8, 31] examined tube/tunnel-handling only via observation, so no restraint was applied to animals in Hurst *et al*.’s studies. In scientific research, most experiments do need to obtain more information from mice than behavior, therefore restraint is needed. This restraint, even if it was just performed once a week, may have had a greater influence than predicted.

Another explanation could be the difference in frequency and duration of handling in the different studies. Hurst and West handled CD-1, BALB/c and C57BL/6N mice only for 9 days and reported a significantly improved performance of non-aversive handled mice in behavioral testing [8, 31]. Novac *et al*. reported an absence of handling effects in their study handling CD-1 mice daily for 15 weeks [35], while we handled mice 4 times per week for 8 weeks and did see, contrary to them a handling-effect but not as large as shown by Hurst *et al*. [8]. It is known that mice may adapt to experimenter handling [36] thus, the effect of tube-handling in the present experiment should be in between the reported outcomes of the previous studies, which they are. Hence, we may see an effect of mice slightly habituated to handling.

The major consequences of handling were seen for forceps-handled animals. As predicted, group-housed, forceps-handled mice showed increased levels of aggression-related behavior. The number of attacks was significantly higher compared to tail- or tube-handled conspecifics. So far, no effects of forceps handling on aggressive behavior have been reported in the literature, consequently no previous data exist for us to compare our results. However, aggression is a welfare issue for mice and moreover creates additional variability [3, 37]. Interestingly the fecal corticosterone levels, which are thought to reflect stress experienced by the mice, did not show any difference between handling groups. These findings are in contrast with previous studies, which found changes in handling methods associated with changes in plasma corticosterone concentrations [38, 39].

We had expected forceps-handled mice to become more sensitive to pain, as this method is from an anthropocentric point of view the most painful one. Forceps-handling implied lifting the animals 4 times a week by forceps on the base of their tail and even if the distal part of the forceps is edged in plastic, it would seem to have an impact on pain perception. The results of this test, although not significant, were interesting as they contradicted our original hypothesis. Forceps-handled animals stayed the longest time on the plate, longer than mice handled with both other methods. An explanation might be a higher stress-induces analgesia (or hypoalgesia) in the forceps-handled mice, which is a well-known phenomenon [40]. As effective pain assessment is critical in terms of animal welfare and the reduction of variation within experimental group, an influence of other painful procedures on those which are conducted with the ongoing research experiment, should be determined [32]. Routine husbandry procedures therefore should not influence those outcomes.

We used rectal temperature to investigate the distress caused by different handling methods. The body temperature can be measured by a rectal thermometer [41, 42] but this method may increase mouse distress due to handling [43, 44]. Therefore, all animals were handled similarly for rectal temperature measurements. We hypothesized that a difference in the temperature measurement was dependent on the previous, 4 times a week conducted handling method, not on the brief restraint required for the measure. Our experiment demonstrated SIH was altered by handling conditions in forceps-handled mice in the last week of measurement: Their SIH was greater than in the other handling conditions, thus their stress-recovery within the 30 min after being handled was delayed. Though a consistent insertion of the probe is important (Bouwknecht et al recommend 2–2.5 cm [27]) we only inserted approximately 1 cm of the rectal probe as the mice were only 3 weeks old on arrival and we wanted to avoid potential injuries to the animal. As this may have led to unreliable measurements, further investigations of the SIH would be needed to verify our outcome.

The investigation of housing effects on mice is far more established than those of handling. In general, group housing is recommended over single housing [1] to maximize well-being of these social animals [45, 46]. However, separation of co-housed animals is a practical solution if aggression arises, even though it is known that male mice prefer each other’s company over individual-housing [47, 48]. Current research opinions differ concerning the consequences of housing configuration on stress-related parameters. Results of several studies indicate individual housing causes stress to mice resulting in stereotypy, nervousness, or handling difficulties [48]. Furthermore, single housing may affect the animals’ physiological stress response and hypersensitivity against toxins [47, 49]. Contrary to those results, Kamakura et al published in 2016 that single housing caused less stress to mice when compared with group housing [50], underlining their results by urinary corticosterone analysis. Urinary corticosterone levels were hereby decreased in individually-housed mice. Those results are supported by previous studies which led to the same findings [51, 52]. Kappel *et al*. recently published a review paper discussing whether it is in the best interest of male mice to be housed together in groups or alone [53], concluding that housing is highly context dependent.

The present study demonstrated several differences in animals based on housing condition. The body weights measured after 2 months clearly differed between group- and single-housed mice with single ones being lighter. Stress can influence the food intake and therefore the body weight, whereby some stressors lead to a decrease, but others have the opposite effect [54–57]. In our results, the weight differential seen could be due to nourishment competition in group-housed mice resulting in an increase in food intake compared to singly-housed mice, or singly-housed mice were stressed by isolation.

However, looking at the results of the fecal corticosterone metabolite analysis, no adverse effect of single housing is obvious. Instead increased FCM levels in group-housed mice were found. Corticosterone, a glucocorticoid which is released from the adrenal cortex in response to a stress stimulus, is a well-established stress marker in research [29, 52]. Even though it is most commonly detected in blood plasma, blood sampling itself as an invasive technique is known to be stressful, especially for small animals [29, 51]. Based on these facts we chose to collect fecal samples for measuring corticosterone metabolites instead, which is a non-invasive technique able to measure prior substantial stress [58]. The measured FCM levels in our singly-housed mice were decreased compared to group-housed animals perhaps indicating less stress compared to their group-housed conspecifics.

This conclusion can be underlined regarding the findings in the nest building test. Mice in both housing conditions improved their initial nest building performance during the course of the experiment. However, scoring after 24 h revealed a nest-building improvement only in singly-housed mice. As nest building behavior is an indicator of well-being in mice and reduced by stress [13, 59], group-housed mice seem to be more stressed in our study. To determine the position of an individual inside the dominance hierarchy of group-housed animals, aggressive interactions are an inevitable consequence, causing stress in animals involved in the agonistic encounter and bystanders. This could be an explanation for the previous results. However, the complexity of nests is of less impact in group-housed mice since they may compensate for lower nest qualities by physical contact [60]. Therefore the cold stress described by Gaskill *et al*. [60] could have led to respective differences in behavior since the impact of nest building in singly-housed mice is higher.

Looking at the data of the SIH, the single-housed mice experienced a single event of stress induced hyperthermia in the 10^th^ week, which is not acutely evident anymore in the following week. SIH has been described in group- and single-housed conditions in several studies [27, 61, 62]. Due to our rectal temperature probe insertion of only 1 cm rather than the recommended 2–3 cm we could have caused some unreliable measurements.

In the RIT singly-housed mice showed more thrust- and aggressive behavior towards the intruder, whereas the distribution of attacking the intruder-males was equal for singly- and group-housed animals. Our results are therefore not consistent with previous findings which state that group-housed male mice are less likely to attack the intruder mouse [63]. We had to modify the standard protocol due to our experimental design; we could not house our residents individually in company with females before testing, as we wanted to examine the effect of handling conditions on behavior in different housing conditions. Additionally, our intruders were all housed singly to prevent a development of a social hierarchy, which might confound results by researchers selecting a mouse at different places in that cage’s dominance hierarchy, possibly influencing the resident’s reaction. In previous studies we consulted, intruder mice were always group-housed. The predicted outcome of singly-housed mice being more aggressive was only partially confirmed, as mice in both housing conditions did not differ in their attack latency, but singly-housed mice did show more aggressive behavior in total.

Another housing effect was detected in the OF, where group-housed mice exhibited fewer anxiety-related spatial movement patterns, supporting previous research on the effect of housing conditions on activity and anxiety behavior in mice [64]. In the sNO assay, the introduction of the caged male mouse, which allowed olfactory, acoustic and limited physical contact (but no biting), revealed no differences in behavior based on housing.

We used mice ordered from commercial breeders by age to study intermale aggression, therefore we probably mixed non-littermates, even if it is known that grouping littermates decreases aggression [21]. This approach was chosen to investigate aggressive behavior under most realistic conditions, because housing only littermates together in cages is mostly not achievable due to the high number of mice required in experiments.

Even though our aim was to study aggression in male mice, agonistic interactions among all males were generally low in our study. An explanation might be that we kept the mice in stable groups from weaning throughout the experiment. Another approach to explain the low aggression could be the way we investigated for biting wounds. Even though bite wounds are difficult to see through fur [65] we chose to use only visual examination as further handling of the mice would be an additional source of stress. It is likely we missed smaller injuries on the animals. Another explanatory approach might be found in the environment of our experimental setting. Only male mice were investigated and therefore they were kept in the experimental room without any female mice. Might the absence of females and consequently their pheromones might have led to the decreased levels of intra-cage aggression? The question of whether the presence of female mice influences male agonistic interactions has so far not been tested. A further investigation of this aspect, *i*.*e*. redoing the experiment with female mice in the husbandry room could be a valuable addition to the mouse aggression literature.

### Conclusion

Our findings assessing the method of handling on aggression-associated parameters in C57BL/6NCrl mice do have important implications for common handling practices. Lifting mice up by their tails with forceps appears to stimulate aggressive behavior within groups of familiar adult mice more than lifting them up with fingers or in a tube. Consequently, this method influences behavioral assessments of aggression. Confirming the recommendations of Hurst *et al*. (18), tube-handling should be applied when minimization of anxiety in experimental mice is desired. Moreover, since group vs. single-housing significantly influenced some of our test results, these maintenance conditions should be considered carefully when planning or analyzing an experiment. The findings of the present study should be kept in mind when forceps-handling is the standard handling procedure (*i*.*e*. IVC cage changing), especially if the parameters being investigated in the course of an experiment are pain-, stress- or behavior-related.

## Supporting information

S1 Fig Scoring sheet. For clinical assessment mice were observed once a week after weighing for clinical signs which might include one or more of the following: weight loss, wounds, barbering sings. Once the mice have been scored grade 2 they were observed more frequently (once a day).
(PDF)
