# Expression of Concern: Effect of three different forms of handling on the variation of aggression-associated parameters in individually and group-housed male C57BL/6NCrl mice

**DOI:** 10.1371/journal.pone.0294388

**Published:** 2023-11-09

**Authors:** The PLOS ONE Editors

After publication of this article [1], concerns were raised about missing data, reporting of the statistical analysis performed as part of the work, and the values in Table 1.

In discussing these concerns with the authors, updated values for Table 1 were provided. The values for Effect size (Cohen’s d) for weight, week 8 (g); temperature difference week 7 (°C); and temperature difference, week 8 (°C) should be updated to -0.57, 0.55, and 0.10, respectively. An updated table is provided in Table 1. It was also noted that the email address for the corresponding author was no longer correct. The correct email address for Sinja Mertens is sinja.mertens@web.de.

As part of its review into this matter, the journal requested additional details regarding the statistical analysis performed, in addition to a request related to data availability. The following was requested:

- Further details on how repeated-measures analysis was performed, for example indicating whether this was done using linear mixed effect models or MANOVAs.
- Whether one-way or two-way ANOVAs were performed and if the latter, whether a type 1 or type 2 ANOVA was performed.
- Further details on how Cohen’s d was calculated.
- All data necessary to replicate the study’s findings be made available in a public data repository.

The authors did not respond to these requests. A spreadsheet with data associated with the study was provided in prior communications, but the journal was unable to obtain permission from the authors to publish this file.

In light of the unresolved concerns relating to the statistical analysis performed and data availability, the *PLOS ONE* Editors issue this Expression of Concern.

**Table 1:** Updated statistical results concerning the factors ‘housing’ and ‘handling’.

				Effects/ interactions			Post hoc test (Tukey)			Effect size (Cohen’s d)			
	Test	Parameter	Statistical test	Housing	Handling	Housing x handling	Hand vs. forceps	Hand vs. tube	Forceps vs. tube	Housing	Hand vs. forceps	Hand vs. tube	Forceps vs. tube
Clinical parameters	Body weight	weight, week 8 (g)	ANOVA	*p = 0.039, F = 4.43, df = 1	n.s.	n.s.	n.s.	n.s.	n.s.	-0.57	-0.07	0.02	0.09
	Temperature difference	temperature difference, week 7 (°C)	ANOVA	*p = 0.046, F = 4.14, df = 1	n.s.	n.s.	n.s.	n.s.	n.s.	0.55	-0.49	-0.45	0.04
		temperature difference, week 8 (°C)		n.s.	**p = 0.009, F = 4.99, df = 2	n.s.	**p = 0.01	n.s.	p = 0.059	0.10	-0.82	-0.21	0.67
	Stress induced hyperthermia	temperature difference, week 8 (°C)	Chi-squared	n.s.	*p = 0.028		*p = 0.019	n.s.	*p = 0.019				
	Blood glucose	Blood glucose, week 8 (mmol/L)	ANOVA	n.s.	n.s.	**p = 0.005, F = 6.5, df = 2	n.s.	n.s.	n.s.	-0.13	0.33	0.36	0.09
	Fecal corticosterone metabolites	corticosterone, week 7 (pg/well)	ANOVA	**p = 0.005, F = 8.98, df = 1	n.s.	n.s.	n.s.	n.s.	n.s.	-1.00	0.20	-0.50	-0.56
		corticosterone, week 7 (ng/0.05 g feces)		**p = 0.005, F = 8.98, df = 1	n.s.	n.s.	n.s.	n.s.	n.s.	-1.00	0.20	-0.50	-0.56
		corticosterone, week 7–3 (pg/well)	ANOVA, repeated measurements	**p = 0.002, F = 10.79, df = 1	n.s.	n.s.	n.s.	n.s.	n.s.				
		corticosterone, week 7–3 (ng/0.05 g feces)		**p = 0.002, F = 10.77, df = 1	n.s.	n.s.	n.s.	n.s.	n.s.				
Behavioral analysis	BS of aggression-associated parameters	cages showing aggression, week 5–8, attack yes/no	Chi squared	/	**p = 0.002		**p = 0.004	n.s.	*p = 0.02				
	Nest-test	quality, week 3, 5 h scores	Mann-Whitney-U or Kruskal-Wallis	*p = 0.025	n.s.		n.s.	n.s.	n.s.				
		quality, week 4, 5 h scores		**p = 0.01	n.s.		n.s.	n.s.	n.s.				
		quality, week 6, 24 h scores		**p = 0.01	n.s.		n.s.	n.s.	n.s.				
		quality, week 8, 24 h scores		*p = 0.044	p = 0.061		*p = 0.033	n.s.	n.s.				
		quality, week 3–8, 5 h scores		***single: p < 0.001 ***group: p < 0.001	***hand: p < 0.001 *forceps: p = 0.037 ***tube: p = 0.001	*SH p = 0.011 SF: n.s. *ST: p = 0.013 ***GH: p = 0.001 GF: n.s. GT: p = 0.068							
		quality, week 3–8, 24 h scores		*single: p = 0.027; group: p = 0.102	hand: p = 0.32; forceps: p = 0.145; *tube: p = 0.049	SH: n.s. SF: n.s. *ST: p = 0.043; GH: n.s. GF: n.s. GT: n.s.							
	Openfield	distance to walls 0–10 min (cm)	ANOVA	**p = 0.006, F = 8.76, df = 1	n.s.	n.s.	n.s.	n.s.	n.s.	-0.89	-0.15	0.54	0.69
		time in center 0–10 min (s)		**p = 0.0011, F = 7.2, df = 1	n.s.	n.s.	n.s.	n.s.	n.s.	0.93	0.12	-0.38	-0.56
	Social Novel-Object test	distance moved 0–5 min (cm)	ANOVA	n.s.	n.s.	*p = 0.035, F = 3.75, df = 2	n.s.	n.s.	n.s.	-0.02	0.15	0.51	0.39
		velocity 0–5 min (cm/sec)		n.s.	n.s.	*p = 0.036	n.s.	n.s.	n.s.	-0.05	0.15	0.50	0.37
		time in center 0–10 min (s)		*p = 0.043, F = 4.4, df = 1	n.s.	n.s.	n.s.	n.s.	n.s.	-0.70	0.15	-0.03	-0.15
	Light/dark box test	exit latency (s)	Mann-Whitney-U or Kruskal-Wallis	n.s.	*p = 0.031		n.s.	**p = 0.007	n.s.				
		tail rattling (s)	Mann-Whitney-U or Kruskal-Wallis	*p = 0.036	n.s.		n.s.	n.s.	n.s.				
		aggressive bite (s)		*p = 0.043	n.s.		n.s.	n.s.	n.s.				
		attack (s)		*p = 0.024	n.s.		n.s.	n.s.	n.s.				
		fighting (s)		*p = 0.016	n.s.		n.s.	*p = 0.038	n.s.				

n.s = no significance. Identification of overall effects of the factors ‘handling’ and ‘housing’ as well as interaction effects. Asterisks indicate the level of significance

(* p ≤ 0.05;

** p ≤ 0.1;

*** p ≤ 0.001). Data is split in ‘clinical parameters’ and ‘behavioral analysis’ for better overview. Non-parametric tested parameters are highlighted in grey. The effect size (Cohen’s d) was calculated for normal distributed data analyzed with ANOVA. Parameters not appearing in this table revealed non-significant results.
