# Supplementary material for: Effect of three different forms of handling on the variation of aggression-associated parameters in individually and group-housed male C57BL/6NCrl mice
Source: PLoS One. 2019 Apr 12;14(4):e0215367. doi: 10.1371/journal.pone.0215367 (PMC6461241; doi:10.1371/journal.pone.0215367)
Supplement: S1 Fig — For clinical assessment mice were observed once a week after weighing for clinical signs which might include one or more of the following: weight loss, wounds, barbering sings. Once the mice have been scored grade 2 they were observed more frequently (once a day). (PDF) [file pone.0215367.s001.pdf]

Project license number: G-154/17
experimenter: _______________
date: _______________________

week: ___
starting time: _______________
ending time: ________________

**Cage Number/Animal Number**	**_______/_______**
Body weight (g):	last week ______<br>current weight ________
	Score: 0: no weight loss<br>1: 0 – 5 % weight loss<br>2: 5 – 15 % weight loss<br>3: 15 – 20 % weight loss
Wounds (n):	
	Score: 0: none<br>1: superficial or < 1 cm<br>2: deep or 1 – 1.5 cm<br>3: ulceration or > 3 wounds or > 1.5 cm
Barbering:	
	Score: 0: no hair loss<br>0,5: < 30% hair loss<br>1: < 50% hair loss<br>1,5: < 75% hair loss<br>2: 100% hair loss
Total Score:	

Euthanasia guidelines: 1. score of 3 in 1 category
2. score of 2 in 2 categories
3. total score of ≥ 4.5
